# Biomarkers in clonal haematopoiesis of indeterminate potential (CHIP) linking cardiovascular diseases, myeloid neoplasms and inflammation

**DOI:** 10.1007/s00277-025-06244-x

**Published:** 2025-02-24

**Authors:** Hui Shan Valerie Tan, Haowen Jiang, Samuel Sherng Young Wang

**Affiliations:** 1 Lee Kong Chian School of Medicine Nanyang Technological University, Singapore, Singapore; 2 Cardiology, National Heart Centre Singapore, 5 Hospital Drive, Singapore, 169609 Singapore

**Keywords:** Biomarker, Inflammation, Myeloid neoplasm, Cardiovascular disease

## Abstract

There is increasing evidence that points to ubiquity of clonal haematopoiesis of indeterminate potential (CHIP) especially with rising age. CHIP has been associated with a multitude of inflammatory, cardiovascular and malignant conditions. In this review article, we evaluate the current literature on CHIP and clinical associations with cardiovascular and haematological diseases. We also discuss high risk features of CHIP that predispose to haematological malignancies, as well as further zoom in on the association between clonal haematopoiesis and therapy-related myeloid neoplasm (tMN). CHIP increases risk of atherosclerotic cardiovascular disease and other cardiovascular conditions such as heart failure, arrhythmias and valvular disease. Hematopoietic clones with mutations in TP53 and spliceosome gene U2AF1 in particularly have repeatedly been shown to increase risk for AML. Other factors such as increased clonal size i.e. variant allele fraction (VAF), clonal complexity have also been shown to increase risk for haematological malignancy. In this comprehensive review, we consolidate the most recent advancements in the understanding of clonal haematopoiesis of indeterminate potential (CHIP) and its associations with cardiovascular and haematological disease. This review is also one of the first to focus on potential biochemical markers routinely utilized in clinical practice that may suggest a more sinister progression of CHIP. We hope to provide physicians with a nuanced perspective on the evolving landscape of CHIP, and offering valuable insights into its clinical implications and potential prognostic indicators.

## Introduction

Clonal haematopoiesis of indeterminate potential (CHIP) is defined as clonal expansion of hematopoietic cells due to somatic mutations of myeloid-malignancy associated genes in the blood or bone marrow with variant allele frequency (VAF) > = 2%, without evidence of hematologic malignancy. Growing interest in CHIP stems from its many associations, from increasing inflammation and risk of atherosclerotic cardiovascular disease (ASCVD) to predisposing patients to malignancies including both hematologic and solid neoplasms. However, the clinical utility of CHIP remains in question. While clonal haematopoiesis has been proven to be exceedingly common in individuals especially with growing age, whether it leads to significant outcomes is uncertain - hence the term ‘indeterminate potential’. While the absolute risk of CHIP transformation to a hematologic malignancy is low and most individuals with CHIP remain asymptomatic, specific CH features may predispose to hematologic malignancies including therapy-related myeloid malignancies [1]. Similarly, CHIP has raised significant interest due to its increased risk of ASCVD [2]. Moreover, the mechanisms through which CHIP leads to the different adverse outcomes and how these pathways can be utilized via clinical treatment to improve patient outcomes remains to be studied. Currently, there is also limited feasibility to implementing CHIP clinically given the need for gene sequencing to detect clonal haematopoiesis and determine driver mutation if any. In this review, we aim to describe an overview of CHIP and its major associations, and discuss the utility of CHIP in clinical practice.

### Are all CHIPs equal?

**Fig. 1:**
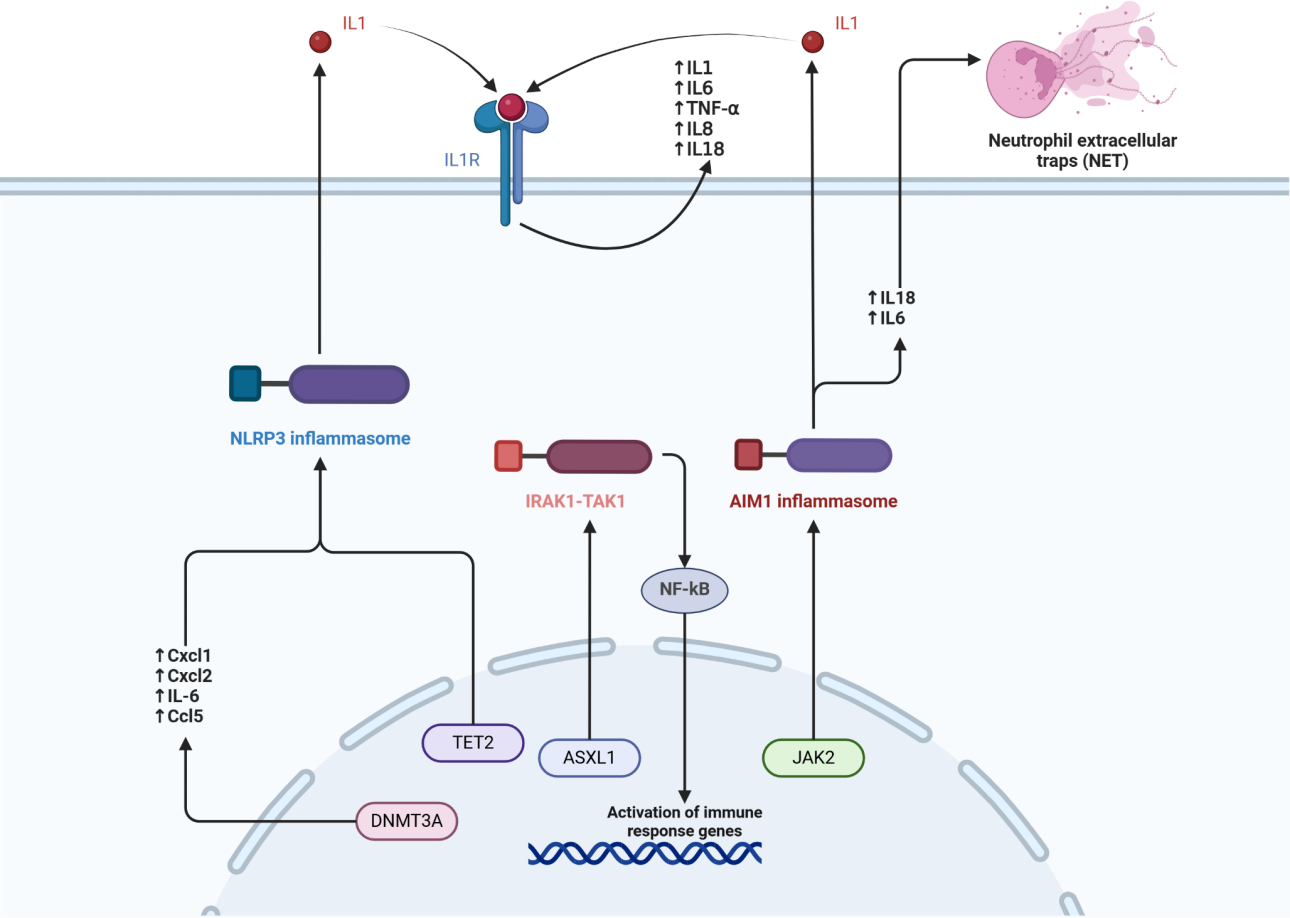
Molecular pathways in clonal haematopoiesis responsible for inflammation and cardiovascular risk for 4 most common CHIP gene mutations and JAK2 mutation

The majority of patients with CHIP have single driver gene mutations, with the most common genes implicated being DNMT3A, TET2, and ASXL1 2. Additional CHIP genes are being discovered yearly, with recently identified genes including ZBTB33, YLPM1, SRCAP, ZNF318 and others [3]. In recent years, several other genes have also become implicated, including JAK2, PPM1D, TP53, SRSF2, SF3B1 amongst others [4]. Different driver gene mutations have varying associations with cardiovascular and haematological disease, likely related to differing mechanisms. However, it appears most CHIP driver mutations lead to a subclinical inflammatory state [5] via a multitude of inflammatory pathways (refer to Fig. 1).

DNMT3A is the most common mutation in CHIP [2]. DNMT3A, which encodes a methyltransferase enzyme, acts as an epigenetic regulator via DNA methylation at CpG sites. Pathogenic loss-of-function DNMT3A mutations encourage self-renewal of hematopoietic stem cells (HSCs), leading to clonal haematopoiesis [6]. It has been shown DNMT3A-mutated HSCs are associated with an inflammatory state, while inflammation itself also perpetuates clonal expansion of DNMT3A-mutated HSCs. Abplanalp et al. found that heart failure patients with DNMT3A-CHIP had increased levels of IL1B, IL6, IL8, the NLRP3 inflammasome, macrophage inflammatory proteins CCL3, CCL4 and resistin [7]. These patients also displayed upregulated expression of immunoglobulin superfamily genes such as CD58, increased expression of T-cell alpha receptor and increased monocyte-T cell interactions. Similarly, in a study of aortic stenosis patients who underwent transfemoral aortic valve implantation (TAVI), it was found that patients with DNMT3A-CHIP displayed pro-inflammatory T-cell polarization [8], Meanwhile, chronic infection has been shown to drive clonal expansion of DNMT3A hematopoietic clones via increased interferon-gamma (IFNγ) signalling [9]. In the same study, Hormaechea-Agulla et al. also demonstrated DNMT3A-mutated human HSCs likewise are driven by IFNγ signalling [9],

TET2 is the second most common driver mutation of CHIP [2]. Haematopoietic clones with TET2 loss-of-function mutations have been associated with increased IL-1B levels via activation of the NLRP3 inflammasome [10]. In mouse models of TET2-CHIP, IL-1B increases levels of pro-inflammatory monocytes/macrophages and promotes self-renewal of TET-2 mutated HSCs via increasing expression of genes responsible for self-renewal [11]. TET-2 mutated HSCs also have resistance against demethylation of transcription factor binding sites important for terminal differentiation that is observed in wild-type HSCs upon IL-1B stimulation, leading to continued self-renewal of these TET-2 mutated haematopoietic clones [11]. TET-2 loss-of-function mutations have been associated with expansion of atherosclerotic plaques in several mouse models as well [10, 11].Fuster et al. demonstrated that increased IL-1B levels was also correlated with increased expression of P-selectin, which promotes endothelial cell adhesion that is vital to early arthrogenesis [10], This possibly explains the proatherogenic mechanism of TET-2 clonal haematopoiesis, TET2 deficiency has also been associated with increased levels of other inflammatory markers like IL-6 [12] and IL-8 [13]. TET2 has been shown to be important for suppression of IL-6 in inflammation resolution [12]. In haematopoietic clones with TET2 deficiency, this pathway is disrupted leading to continued inflammation. Jaiswal et al. have postulated increased levels of IL-8, a CXC chemokine, promotes CXC chemokine and CXCR2 atherogenic interactions at the arterial intima [13].

Increased NLRP3 inflammasome activity in DNMT3A- and TET-2 HSCs play a key role in atherosclerosis, via mechanisms such as increased production of reactive oxygen species (ROS), mitochondrial dysfunction, lysosome rupture and exerting endoplasmic reticulum stress [14]. MCC950, a sulfonylurea derivative and pharmacological inhibitor of NLRP3, has been show to reverse atherogenesis in TET-2 mutant CHIP mouse models [10]. MCC950 was also able to repress IL1B expression in white adipose tissue and subsequent insulin resistance [15], as well as prevent heart failure in TET-2 deficient mouse models [16]. While NLRP3 inflammasome inhibitors like MCC950 appear to be promising, its clinical uses have been limited by reports of hepatotoxicity.

The mechanisms for how ASXL1 (additional sex combs-like 1) mutations lead to inflammation and atherosclerosis have only recently been elucidated. Sato et al. demonstrated that in mice, cells with C-terminally truncated ASXL1 mutant (ASXL1-MT) demonstrated an unexpected non-epigenetic mechanism through loss of inhibition of IRAK1-TAK1, resulting in NF- κB activation [17]. This activation causes activation of the innate immunity and drives systemic inflammation, a possible mechanistic explanation for accelerated atherosclerosis in patients with ASXL1 mutations [17] Furthermore, prior observational studies have also linked ASXL1 mutations to causes of pro-inflammatory states in humans, including smoking and HIV infection, supporting the previous mechanism [18].

Another notable CHIP driver mutation is JAK2 which accounts for approximately 3.2% of CHIP [19]. JAK2 is an important non-receptor tyrosine kinase serving as a downstream signal transmitter for major cytokine receptors [5, 19]. JAK2V617F, a gain of function mutation, particularly is a well-known driver mutation for myeloproliferative neoplasms (MPN) [20] including polycythaemia vera, essential thrombocythemia and myelofibrosis, conditions that increase thrombotic risk. JAK2V617F mutations themselves have also been shown to be prothrombotic, via increasing formation of neutrophil extracellular traps (NET) [20]. A population study involving 10,893 individuals by Wolach et al. also showed that JAK2V617F-CHIP was associated with increased risk of major venous thromboembolism. This highlights the thrombotic propensity of JAK2V617F mutant hematopoietic clones even without other comorbidities such as MPN [20]. Fidler et al. also showed that JAK2 mutant macrophages display increased proliferation, and in this process, increased oxidative damage in the form of mitochondrial reactive oxygen species (ROS), and increased DNA replication stress [21]. Increased ROS levels lead to activation of AIM2 inflammasomes which then increase inflammation via secretion of cytokines such as IL1B [21]. Increased inflammasome activity overall leads to further proliferation of inflammatory macrophages within atherosclerotic plaques and promote necrotic core formation, potentiating atherosclerotic complications such as myocardial infarction [21].

Clonal size is also an important factor in determining cardiovascular risk. The definition of CHIP with a VAF of > 2% was initially established due to limitations in gene sequencing technology [22]. However, it also represents a practical cut-off as recent studies showed that almost all older adults had evidence of clonal haematopoiesis with a VAF of 0.03% which was unlikely to be prognostically significant [23]. However, recent studies have demonstrated that in certain cardiovascular diseases, clonal haematopoiesis with a VAF as low as 0.06% may have prognostic significance [24]. Nonetheless, patients with a larger clonal size (defined as a VAF > 1%) was found to have worse cardiovascular outcomes and a higher risk of myeloid malignancies compared to patients with a VAF of < 1% [25, 26]. Given the diverse range of CHIP phenotypes, it is important to understand which phenotypes are most strongly associated with cardiovascular disease in order to develop effective therapies.

### Associations with cardiovascular disease

#### ASCVDs

CHIP initially gained interest due to its association with all-cause mortality and incidence of coronary artery disease [13]. Independent of traditional cardiovascular risk factors, carriers of CHIP have a risk of coronary artery disease almost twice that of non-carriers, and a risk of myocardial infarction (MI) 4 times that of non-carriers [13]. Patients with larger CHIP clones (defined as a VAF of > 0.01) have an increased risk of CVD events [27]. Animal studies have also demonstrated that Tet2 knockout mice had larger atherosclerotic lesions in the aortic root and aorta than mice with the gene. Since then, interest in CHIP and its role in ASCVD and other cardiovascular diseases has surged. Even in patients with established ASCVD, CHIP is independently associated with an increased risk of ASCVD events and all-cause mortality [26]. This strong association with ASCVD garnered interest in its role as a novel cardiovascular risk factor and possible therapeutic target.

The main mechanism of CHIP causing increased risk of cardiovascular disease is thought to be attributable to chronic inflammation. Loss of function mutations of DNMT3A and Tet2, the most common gene mutations, have been demonstrated to enhance inflammation in macrophages in vitro and generate a distinct adventitial macrophage population in vivo, promoting innate immune cell activation [28]. Inflammatory cytokines IL-1b and IL-6 appear to play a central role in mediating inflammation and subsequently atherosclerosis (i.e. the inflammasome/IL-1/IL‐6 pathway). Individuals with CHIP with a genetic deficiency of IL-6 signalling had an attenuated cardiovascular risk, especially in large CHIP clones, representing a possible therapeutic target [27]. Patients with CHIP due to somatic mutations in TET2 had significantly reduced risk of MACE when given Canakinumab, an anti–IL-1β antibody with anti-inflammatory properties [29]. Interestingly, even in a cohort of patients without known CHIP, Canakinumab also led to a significantly reduced risk of MACE, independent of lipid lowering [30]. In animal studies modelling TET2 or JAK2 CHIP, IL-1b antibodies resulted in fibroblast accumulation and fibrous cap thickening, promoting plaque stability [31]. This highlights the significance of the role of inflammatory cytokines in mediating atherosclerotic processes and represents a possible future target for pharmacological therapies.

However, there may be genes and other factors which may be protective of ASCVD in CHIP patients. In a large prospective study, patients who had developed CHIP with genetic deficiency of IL-6 signalling (by carrying IL6R p.Asp358Ala versus wild-type) was found to have significantly greater reduction in cardiovascular risk compared to without CHIP with genetic IL-6 signalling deficiency [27]. Interestingly, diet has also been shown to be modulating factor between CHIP and ASCVD risk, independent of CHIP status [32]. In patients with CHIP, a healthy diet has been shown to confer an absolute risk reduction in CV risk greater than the general population [32] which again is thought to be due to modulation of systemic markers of inflammation. The complex interplay between various somatic mutations and cardiovascular risk in CHIP is yet to be fully elucidated and should be work for future study.

#### Heart failure

CHIP has also been found to be associated with heart failure, across the spectrum of ejection fractions and aetiologies. Initial trials found a prevalence of CHIP of 18.5% CHIP among patients with ischemic HFrEF [33]. Patients with ischemic HFrEF and CHIP also had significantly worse long-term outcomes compared to patients without CHIP [33–35]. The prognostic significance of CHIP also appears to extend below the historic cut-off of > = 2%. Survival receiver operator curve analyses has demonstrated an optimal cut-off of 0.73% for TET2-driven and 1.15% for DNMT3A-driven CHIP mutations, with a 5-year mortality of 31–32% above cut-off compared to 18–19% for patients with a VAF below optimal cut-off [34].

CHIP has also been associated in patients with non-ischemic HFrEF. In patients without a known history of ASCVD, CHIP predicts the development of HFrEF [36]. CHIP also has prognostic significance in patients with HFrEF regardless of etiology [24, 35, 37]. In cohorts of patients with both ischemic and non-ischemic HFrEF, CHIP is associated with increased risk of HF-death and HF-hospitalization, as well as the development of cardiogenic shock [35, 37]. In patients with dilated cardiomyopathy, CHIP has been associated with increased risk of all-cause mortality independent of LVEF and clonal size [24]. Similarly to patients with ischemic HFrEF, the prognostic significance of CHIP in non-ischemic HFrEF extends below the traditional cut-off of 2% up until a VAF cut-off of 0.06%^24^.

Despite most of the literature focused on HFrEF, recent studies have also shown a significant association to HFpEF. As opposed to HFrEF, the pathophysiology and manifestations are more complex and harder to diagnose [38]. CHIP has been associated as an independent risk factor for incident HFpEF. In a large cohort of 2 racially diverse prospective cohorts of patients, TET2-CHIP was found to be associated as an independent risk factor for development of HFpEF [39]. Patients with HFpEF and CHIP also appear to have a worse cardiac function and prognosis. TET-2 driven CHIP was associated with worsened diastolic dysfunction assessed by E/e’ and E/A ratios, and increased rates of cardiovascular-related hospitalizations in a 5-year period [40]. Mice studies with TET2-deficient bone marrow showed that TET2-mediated CHIP displayed greater features of HFpEF, with increased cardiac hypertrophy, diastolic dysfunction, and cardiac fibrosis [40].

Whilst its association with ischemic heart failure with reduced ejection fraction (HFrEF) can be partially explained by the same processes that mediate atherosclerosis, how CHIP causes non-ischemic HFrEF is less clear. In animal studies with TET2-deficient bone marrow, macrophages accumulate in the myocardium, resulting in inflammation and subsequent fibrosis, eventually leading to a lower ejection fraction [16]. Similarly, inactivation of DNMT3A in macrophages amplifies release of cytokines such as epidermal growth factor-like growth factor, resulting in activation of cardiac fibroblasts [41]. These pathways represent a potential therapeutic target for future therapies, particularly looking at the role of anti-fibrotics in the treatment of heart failure. Recent research has also suggested the existence of a “heart-cancer axis” owing to a close relationship between the pathogenesis of both diseases and multiple complex interaction between malignancies and cardiovascular disease [42]. Heart failure has been associated with increased rate of cancer progression via release of cardiokines, and reciprocally cancer promotes heart failure via muscle wasting and remodelling of the heart [42]. Understanding these mechanisms not only allows us to explain why patients with cancer have increased risk of heart failure and vice versa but also represents potential therapeutic targets for future research.

#### Arrhythmias

CHIP has also been associated with arrhythmias, including supraventricular and ventricular arrhythmias, as well as bradyarrhythmia in a study of UK Biobank individuals without such prior diagnosed conditions [43]. A different study by Lin. et al. investigating rates of incident AF among UK Biobank participants with CHIP found that CHIP with loss of TET2 function in particular was associated with increased incident AF [44]. In this same study, analysis of mouse models with TET2 inactivation found loss of TET2 function inhibits calcium release from the sarcoplasmic reticulum, possibly contributing arrhythmogenic mechanism of TET2 CHIP. TET2 deficient mouse models were also associated with increased activity of NLRP3 (NLR [NACHT, LRR {leucine rich repeat] family pyrin domain containing protein 3) and CaMKII (Ca2+/calmodulin-dependent protein kinase II). A recent East Asian study investigating prevalence of 24 CHIP mutations in AF vs. non-AF healthy subjects found increased incidence of DNMT3A and TET2 mutations among AF individuals, and this was associated with more malignant AF and less favourable clinical outcomes such as heart failure, ischemic stroke and death [45].

#### Valvular disease

CHIP may also be associated with and carry prognostic significance in patients with valvular heart diseases. In a cohort of patients with severe aortic stenosis undergoing transcatheter aortic valve implantation (TAVI), TET2- and DNMT3A-driven CHIP was found to be associated with a profound mortality risk even after successful TAVI [8]. This mortality risk remained even after prolonged follow-up of up to 5 years [46]. Various gene mutations are associated with different mortality risks. Patients carrying TET2-mutations had a significantly decreased survival after TAVR compared to CHIP-negative patients or other gene mutations [46]. Interestingly, it was observed that patients with combined DNMT3A and TET2 CHIP-driver variants have a lower risk of mortality after TAVR in comparison with patients carrying TET2 alone suggesting a protective role of DNMT3A only when present together with TET2 mutations [46]. Whilst it is speculated that the mechanisms are similar to those causing atherosclerosis, mediated via increased circulating inflammatory cells resulting in valve calcification via cell proliferation and expression of matrix metalloproteinases, it is difficult to explain the differing risks associated with different genes [8, 46]. Complex mechanisms and interactions are likely at play which have yet to be described. Furthermore, CHIP has been found to be associated with patients with aortic valve sclerosis (AVS) [47] and was found in high frequency in patients with aortic valve stenosis. Whilst the most frequent gene mutations are DNMT3A- and TET2-CHIP, a large diversity of gene mutations have been implicated. CHIP has also been suggested to affect calcified aortic valve disease progression. Patients with AVS and CHIP had significantly higher calcium scores than patients without CHIP [47]. As TAVI and other transcatheter valve interventions gain popularity, CHIP may represent an important prognostic factor in this growing group of patients.

#### Peripheral arterial disease and other vascular diseases

CHIP is also associated with increased risk of peripheral arterial disease (PAD). In a large cohort of over 50,000 patients with whole-exome sequencing and clinical data from the UK biobank and Mass General Brigham Biobank, CHIP was associated with a 58–66% increased risk for incident PAD [48], with a higher VAF associated with greater risk for PAD. CHIP was also a risk factor for atherosclerosis across multiple vascular beds, with significant associations for aortic aneurysms (HR 1.74, 1.21–2.51), other aneurysms (HR 1.70, 1.20–1.63), and mesenteric ischemia (HR 3.22, 2.01–5.17) [48]. Gene-specific analysis revealed this increased risk was primarily driven by mutations of DNA damage repair genes TP53 and PPM1D [48], consistent with prior reports [49–51]. Animal studies subsequently confirmed the excessive proliferation of p53-deficient plaque macrophages in mice, driving increased atherosclerosis throughout the entire arterial system [48]. There is also evidence that atherosclerosis may accelerate somatic mutations in CHIP, suggesting a bi-directional relationship between the 2 diseases [52].

### Haematological malignancies

CHIP is associated with an increased risk of haematological malignancies and death from haematological malignancies [53], although the absolute risk of progression from CHIP to haematological malignancy is small. In a longitudinal study by Jaiswal. et al. involving eight years of follow up of 3341 individuals, only 4% of subjects with CHIP developed a hematopoietic malignancy [2]. Nonetheless, a case-control study by Desai et al. showed that the sole presence of mutations was associated significantly increased risk of AML development, and this association was significant even after correction for age [54]. Individuals at least 65 years of age had increased OR = 6.19 (*p* < 0.05) compared to those < 65 years old at OR = 4.39 (*p* < 0.05)^54^. In a large biobank of over 40,000 CHIP carriers, CHIP was associated with a 22–47% increased of death from leukaemias compared to the general population [53]. A better understanding of CHIP characteristics that predispose patients to developing hematologic malignancies can allow clinicians to pay close attention to these high risk patients, and implement early interventions as needed.

#### Driver mutation

Naturally, one would first consider the type of CHIP mutation and its relationship to the risk of hematopoietic malignancy. In the abovementioned study by Desai et al., IDH1, IDH2, TP53, DNMT3A, TET2 and spliceosome genes such as U2AF1, SF3B1 and SRSF2 mutations significantly increased risk of progression to AML on multivariable analysis correcting for potential confounders such as age and presence of comutations [54]. This risk was further increased in individuals with more than one variant in DNMT3A or TET2. Abelson et al. also conducted a case-control study of patients with clonal haematopoiesis who developed acute myeloid leukaemia (AML), and also attempted to quantify the risk contribution of different driver mutations for AML. Abelson et al. found that mutations of TP53 and U2AF1 led to greatest risk of progression to AML, with hazard ratios of 12.5 and 7.9 respectively [55]. Notably, DNMT3A and TET2 driver mutations were the most common among both the pre-AML cases and the control group and were associated with decreased risk of AML development [55]. Interestingly, all individuals with TP53 (*n* = 21), and IDH1 or IDH2 (*n* = 15) gene mutations developed AML in the study by Desai et al. [54] However, it is difficult to extrapolate such a finding to the general population given the study by Desai et al. only included female subjects.

#### Clonal complexity

It also appears that increased clonal complexity is also associated with progression to AML. In the study by Desai et al., presence of co-mutations showed increased odds ratio for AML at 9.01 (95% CI 4.1–21.4, *p* < 0.05) [54]. The most common co-mutations among the AML cases were DNMT3A with TET2, DNMT3A with SRSF2, TET2 with SRSF2, and IDH2 with SRSF2 54], while most of the controls only had sole driver mutations present. Notably, DNMT3A and TET2 mutations were shown to be mutually exclusive in the control group. Data from large biobanks have also suggested driver mutation zygosity as an important factor for predicting CHIP transformation and disease severity [56].

#### VAF

With regards to CHIP transformation to hematologic malignancy, another important CHIP feature to consider is size of the hematopoietic clone. In the above mentioned study by Jaiswal et al., subjects with at least VAF 0.10 hematopoietic clones had increased hazard ratio of nearly 50 for developing haematological malignancies [2]. However more recently, studies have shown that clonal haematopoiesis not amounting to CHIP with VAF < 0.02 are also associated with increased risk of haematological malignancies. In the aforementioned case-control study by Abelson et al., clonal haematopoiesis involving the genes TP53, IDH1 and IDH2, SRSF2, SF3B1 and U2AF1 with VAF > 0.01 was shown to increase risk of AML development [55]. Similarly, Desai et al. found that VAF of more than 1% in mutations of IDH2, TP53, DNMT3A, TET2 and spliceosome genes was associated with increased risk of AML development, though VAF > 10% were associated with even greater risk [54]. Another 2019 case-control study by Young et al. found that clonal haematopoiesis with VAF of at least 0.01 in any variant was enough to increase risk of AML development [25]. The study investigated 54 leukaemia associated genes and common mutations among their study cohort included DNMT3A, TET2, ASXL1, BCORL1, and TP53.

Currently, the definition of CHIP requires hematopoietic stem cells (HSCs) with somatic mutations with VAF of at least 2%. However as mentioned above, it is worthwhile to note that this is an arbitrary threshold derived largely from the technical limitations of next generation sequencing techniques with no clinical significance to the figure. Increasingly, age-related clonal haematopoiesis (ARCH), that is defined as gradual, clonal expansion of HSCs carrying specific, disruptive, and recurrent genetic variants, in individuals without clear diagnosis of hematological malignancies [57], is also associated with increased risk of haematological malignancies as mentioned above. Hence we propose that it may be more worthwhile to classify clonal haematopoiesis into varying degrees of severity based on VAF, such as mild, moderate and severe, over terms such as ARCH and CHIP.

#### Therapy related myeloid neoplasms

Clonal haematopoiesis has also been correlated with increased risk of therapy-related myeloid neoplasms (tMN). New understanding of the mutational landscape in tMN has led to a paradigm shift in the postulated pathophysiology of tMN. While it was previously thought that tMN are induced via mutagenic effects of malignancy cytotoxic therapy, there is increasing evidence that majority of tMN driver mutations are derived from pre-existing clonal hematopoietic cells. A study by Wong et al. is one of the first to demonstrate this; They identified four cases of therapy related acute myeloid leukaemia (t-AML) and myelodysplastic syndrome (t-MDS) whereby TP53 mutations were detected in peripheral blood samples or bone marrow 3–6 years before tMN diagnosis [58]. Bolton et al. studied 35 individuals who developed tMN and had available paired blood samples analysed at CH and tMN diagnosis. 94% of these tMN cases already had the tMN disease-defining mutation present at time of CH diagnosis [59]. 91% of cases also further acquired somatic mutations in genes known to be late drivers of myeloid malignancy (such as FLT3, KRAS, NRAS), and chromosomal aneuploidies, driving transformation to tMN [59]. Soerensen et al. compared 36 cases of patients with non-myeloid disease who were treated with autologous stem cell transplantation (ASCT) and subsequently developed tMN with 36 control patients who also underwent ASCT but did not develop tMN [60]. Clonal haematopoiesis at time of ASCT was associated with increased likelihood of progression to tMN (OR 5.9 *p* = 0.003) [60]. Notably, all patients less than 50 years old with clonal haematopoiesis detected at ASCT developed tMN [60]. One patient who had NGS analysis performed at ASCT and tMN diagnosis revealed expansion of pre-existing hematopoietic clones driven by SRSF2 mutation, from VAF 0.005 to 0.45. Interestingly, clonal size of TP53, TET2, ASXL1 gene mutations that were also present at time ASCT decreased instead [60]. Putting these pieces of information together, it appears that genotoxic stress exerted by cytotoxic malignant therapy leads to selection of HSPC and together with additional acquired somatic mutations, lead up to development of tMN. Additionally, case-control studies by Gillis and Takahashi et al. found that clonal haematopoiesis present at primary cancer treatment increases risk of tMN by ten times [61, 62]. In a retrospective case-control study by Takahashi et al. that compared 14 cases of patients treated for malignancy that developed tMN to 54 age-matched controls matched who had underwent prior lymphoma treatment, 71% of cases (10 out of 14) had CHIP compared to only 31% of controls [62].

There are several proposed mechanisms through which cytotoxic therapy leads to selection of pre-existing hematopoietic clonal populations. Link and Walter et al. have postulated that mutant hematopoietic stem cells (HSCs) have a selective advantage in a cytotoxic therapy environment [63]. This is especially so for mutant HSCs with TP53 related mutations. Indeed, several studies have shown that HSCs with DNA damage response (DDR) gene mutations, particularly in TP53 and PPM1D undergo clonal expansion after cytotoxic exposure [64–66]. In-vivo mouse models that have undergone cytotoxic exposure via chemotherapy also show that TP53-mutated cells have greater expansion than wild-type cells [58].

### Clinical utility of CHIP

**Fig. 2:**
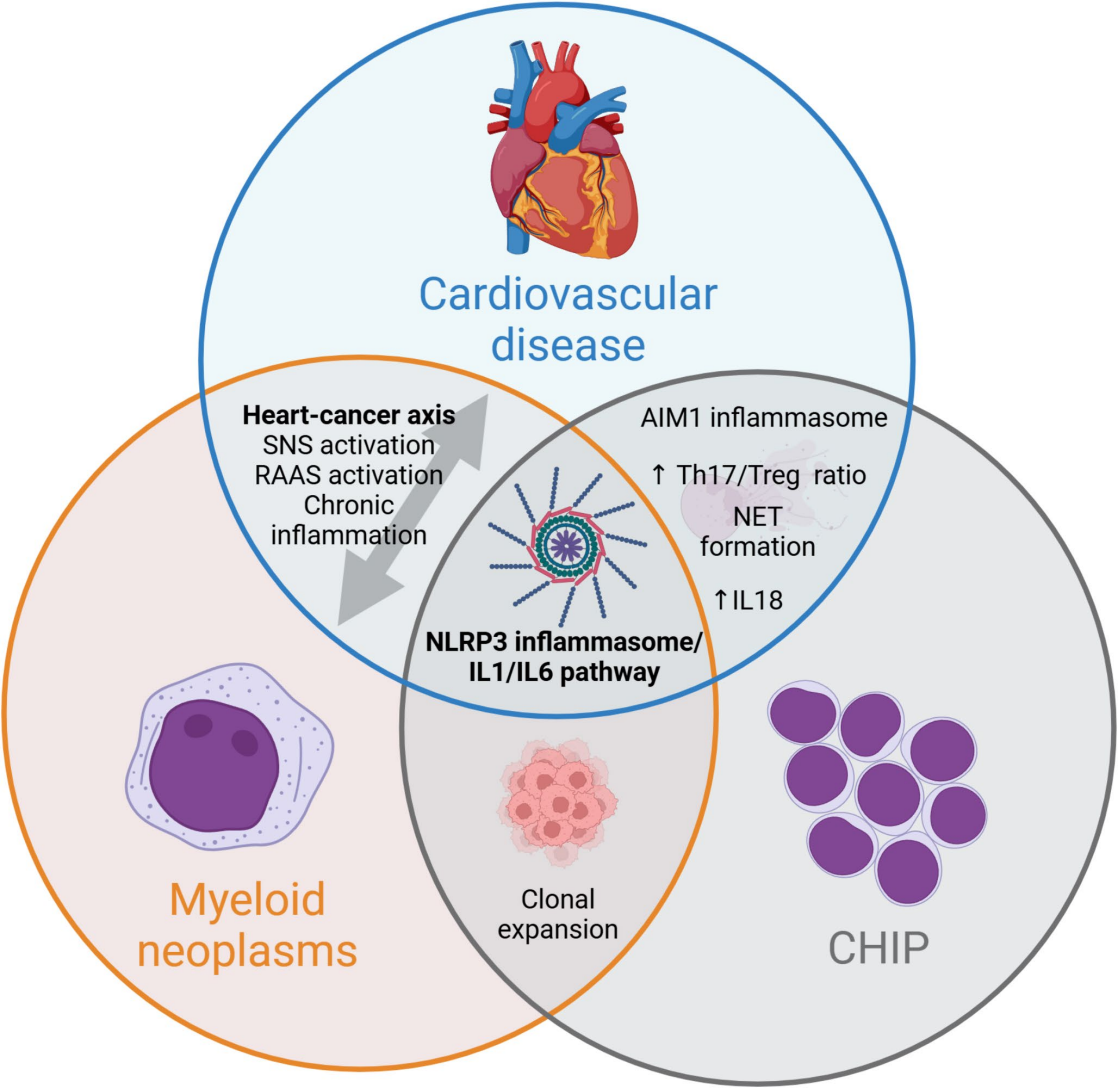
Overlaps in Features between Clonal Haematopoiesis of Indeterminate Potential, Myeloid Neoplasms and Cardiovascular Disease

While CHIP clearly has significant associations and prognostic implications with a wide range of cardiovascular and haematological diseases (Fig. 2), several factors limit its current use in clinical practice. CHIP requires the use of gene sequencing which is both costly and not readily accessible at present, making it impractical as a routine blood test. More importantly, CHIP is an increasingly common finding as patients age. Almost all patients above the age of 50 have evidence of clonal haematopoiesis with a VAF of 0.03%, and 10–20% of patients aged 70 or older harboured CHIP [2, 67]. With so many patients harbouring CHIP mutations, more specific methods are needed to stratify cardiovascular risk. Several biomarkers have been shown to be associated with CHIP and ASCVD and may have clinical utility. High-sensitivity C-reactive protein (hs-CRP) is a biomarker which has been used to stratify cardiovascular risk in patients [68]. Recent data has found a significant association between CHIP and hs-CRP in CAD cohorts but not in non-CAD patients [69]. Interestingly, other studies did not find an association between CHIP and hs-CRP but did find associations with other inflammatory markers, specifically IL6 and IL1 [27]. This has been postulated to be because hs-CRP is not as specific for CHIP as other inflammatory biomarkers [27]. Nonetheless, this suggests a role for the use of additional inflammatory biomarkers as risk stratifiers in patients with CHIP. See Table 1.

**Table 1:** Biomarkers under clinical/preclinical evaluation for CHIP/inflammation associated with CHIP/ myeloid neoplasm associated with CHIP

Biomarker	Utility in CHIP	Limitations
High-sensitivity C-reactive protein (hs-CRP) [69]	Elevated levels may predict CAD	Some studies did not find association between CHIP and hs-CRP; not as specific to CHIP as other inflammatory markers
Cholesterol, triglycerides [4, 76]	JAK2 CHIP was associated with decreased total cholesterol and LDL-C despite elevated CAD risk	Large scale studies show no association between other types of CHIP and lipid levels
TNF-alpha, IL1, IL6, IL-18 [27, 77]	Elevated levels suggest inflammation and may predict CAD; More specific than hs-CRP	Not routinely tested
Red Blood Cell Distribution Width (RDW), Mean corpuscular volume (MCV) [78]	Increased RDW and MCV is associated to transformation to AML	

A raised red cell distribution width (RDW) has also been associated with risk of clonal haematopoiesis transformation to AML. Abelson et al. analysed RDW trends of 875 individuals with AML that they identified from the Clalit database [70]. Their analysis revealed RDW is raised in the years leading up to AML diagnosis [55]. Other blood count factors that also increased AML risk include decreased red blood cell, white blood cell, platelet and monocyte counts, though usually hovering above the lower limit of normal [55]. Bolton et al. also found RDW as a significant risk factor for AML on univariate analysis [59]. However, raised RDW has also been correlated with inflammatory states, cardiovascular disease and malignancies [71]. While RDW in CHIP might not be a specific marker for inflammation, ASCVD or malignancy, it may remain relevant as a maker for increased risk of adverse outcomes.

One potential area where CHIP may be clinically significant is in a subset of patients without standard modifiable cardiovascular risk factors (SMuRF-less patients) [72]. An increasing proportion of patients presenting with MI do not have SMuRFs (smoking, diabetes mellitus, hyperlipidaemia, hypertension), rising from 11% in 2006 to 27% in 2014 [73]. There is much uncertainty regarding treatment in this growing group of patients given the lack of risk factors to target. The identification of CHIP in this group of patients may allow treatment with novel anti-inflammatory agents to modify CAD risk [27, 29]. However, testing for CHIP requires whole-exome sequencing (WES) which may be costly and should not be done routinely. The decision to test for CHIP in this group of patients requires the exclusion of other risk factors for SMuRF-less CAD and counselling on the genetic implications of testing [72]. More data is also required on the utility of monitoring CHIP as a therapeutic target in SMuRF-less patients.

Given the increasing use of molecular assays and the potential of CHIP as a risk modifier for CVD, haematological malignancies, and other diseases, multi-disciplinary CHIP clinics [74] and biorepositories [75] are being set up. Future data from these clinics and biorepositories may help answer outstanding questions and guide overall management in this emerging patient population.

## Conclusion

CHIP is significant as a risk factor not only for haematological malignancies, but also as a novel cardiovascular risk factor particularly in patients without standard modifiable risk factors. Whilst its clinical utility is limited at the moment, rapid advances in gene sequencing technology may see CHIP being worked its way into standard clinical practice in the near future.

## Data Availability

No datasets were generated or analysed during the current study.
